# Successful Treatment of Subungual Warts With Pulsed Dye Laser: Report of Four Cases

**DOI:** 10.1111/jocd.16756

**Published:** 2025-01-21

**Authors:** Shuang Lyu, Zhenhua Yue, Huimin Zhang, Xi'an Fu, Hong Liu, Furen Zhang

**Affiliations:** ^1^ Hospital for Skin Diseases Shandong First Medical University Jinan Shandong Province China; ^2^ Shandong Provincial Institute of Dermatology and Venereology Shandong Academy of Medical Sciences Jinan Shandong Province China

**Keywords:** laser therapy, pulsed dye laser, subungual warts

To the Editor,

Warts are caused by the human papilloma virus (HPV) and have a considerable impact on quality of life due to pain and embarrassment [1]. Treatment of warts requires multiple destructive procedures, but results are often unsatisfactory. Subungual warts affecting the nail folds can be painful and interrupt daily activities. Destructive treatments require disruption of the nail plate to access the lesion, increasing the risk of injury to the subungual wedge, underlying bone, or nail matrix [2]. Finding less invasive and painless therapies for subungual warts is a challenge for clinicians. Conventional methods include drawbacks such as scar development, a lengthy recovery period, and a high recurrence rate; they may also result in nail plate damage, severe pain, and even nail atrophy [2]. In contrast, pulsed dye laser (PDL) has a low incidence of pain, with only temporary pain and residual hyperpigmentation, making it a safe, tolerable and relatively effective treatment [3, 4]. We present four cases of subungual warts treated with PDL.

## Report of Cases

**FIGURE 1:**
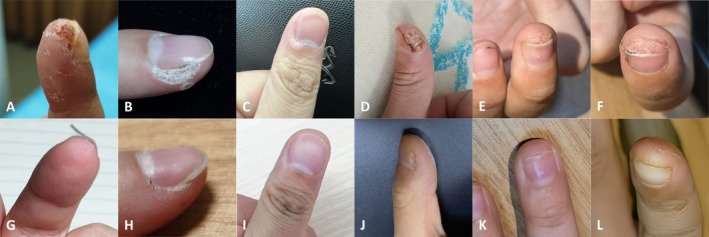
Clinical images before PDL treatment and at final follow‐up. Patient 1: A, G; Patient 2: B, H; Patient 3: C, I; Patient 4: Right thumb (D, J), middle finger (E, K) and left pointer finger (F, L).

Four patients had subungual warts: one female and three males aged 9–40. All four patients had previously undergone cryotherapy, which caused significant pain and was ineffective. The patients were treated with PDL (595 nm, Candela, Israel). Cases 1–4 are presented in Table 1. Clinical pictures were collected before and after each therapy and were followed up (Figure 1). After cessation of treatment, the lesions achieved long‐term remission without nail damage and did not recur during the follow‐up period.

**TABLE 1:** Cases introduction.

Patient	1	2	3	4
Age (years)	25	31	40	9
Disease course	1 years	4 months	3 months	3 years
Gender	F	M	M	M
Position	Left index	Right index	Left thumb	Right thumb, middle finger and left pointer finger
Parameters	21 J/cm^2^, 3 ms	21 J/cm^2^, 3 ms	21 J/cm^2^, 3 ms	23 J/cm^2^, 3 ms
(energy, pulse width)				
No. of sessions	2	4	4	8
Interval^a^	1 week	3–4 weeks	4 weeks	2 weeks
Follow‐up (months)	20 months	3 months	4 months	5 months
Previous treatments (failed therapy)	Cryotherapy	Cryotherapy	Cryotherapy	Cryotherapy
Adverse reaction	Immediate pain	Immediate pain	Immediate pain	Immediate pain

^a^ Interval: time between each session of treatment.

## Discussion

Warts have been found to respond favorably to PDL [5]. PDL's therapeutic techniques involve the breakdown of the wart‐supplying capillaries, which results in host cell death. It can also trigger an immunological response and cause IL‐2 and IL‐4 to be upregulated within the lesion. Furthermore, PDL destroys the virus itself due to its susceptibility to heat [3, 4, 5].

The type of warts, laser parameters, number of treatment sessions, and treatment intervals are associated with the success rate of PDL treatments [4, 5]. A retrospective study treating 227 warts patients with PDL found that up to 6 sessions with intervals of 3–4 weeks apart and a fluence of 12.5–15.0 J/cm^2^ had the best efficacy [5]. Another study showed that higher success rates were associated with higher flow settings (9.5 J/cm^2^) and also an increased number of sessions (up to 6) at 3–4 week intervals [4]. Park et al. have reported that there was no significant difference in lesion clearance between 2‐ and 3‐week treatment intervals [3]. Our patient 1 was healed after just two treatments with a one‐week gap, indicating that shorter treatment intervals could be more effective and reduce the number of sessions. The mean length of treatment in our patients was 4.1 months and the mean number of sessions was 4.5. Notably, patient 4 received 8 treatments, which may be attributed to individual differences, disease duration, and lesion thickness.

PDL appears to eliminate warts only while sparing the surrounding nail plate, causing transient pain similar to that of a rubber band snapping [6]. Our patient 4 was a 9‐year‐old boy who fully tolerated the pain of PDL treatment. PDL is a relatively safe procedure with acceptable side effects [3], produces satisfactory results in cosmetically sensitive areas, and has little effect on daily activities.

The high cost of PDL and the requirement of multiple treatments may result in patients or clinicians choosing it less frequently. However, due to the special location of subungual warts, traditional cryotherapy can be affected by the nail and significantly painful. In conclusion, PDL is an option for subungual warts, especially in children who cannot tolerate pain. This report illustrates the advantages of PDL for the treatment of subungual warts, provides references for treatment parameters and frequency, and provides evidence on effectiveness and safety. However, in clinical practice, attempts have been made to combine it with other treatment modalities (e.g., superficial X‐ray therapy, photodynamic therapy or cryotherapy) to improve efficacy and reduce recurrence rates. Further studies with larger sample sizes are still needed.

## Author Contributions

Conceived by Furen Zhang, Hong Liu and Xi'an Fu. Collected photographs by Zhenhua Yue and Huimin Zhang. Written and edited by Shuang Lyu and Xi'an Fu.

## Ethics Statement

Patients provide informed consent, including treatment and disclosure of case details and images.

## Conflicts of Interest

The authors declare no conflicts of interest.

## Data Availability

The data that support the findings of this study are available on request from the corresponding author. The data are not publicly available due to privacy or ethical restrictions.
